# Exploring the S-shaped relationship between triglyceride-glucose index and serum uric acid levels in individuals with osteoporotic fracture

**DOI:** 10.3389/fendo.2025.1639818

**Published:** 2025-10-24

**Authors:** Xiao-jie Zhou, Min-zhe Xu, Ya-qin Gong, Jian Jin, Ke Lu, Chong Li

**Affiliations:** ^1^ Department of Orthopedics, Affiliated Kunshan Hospital of Jiangsu University, Suzhou, Jiangsu, China; ^2^ Kunshan Biomedical Big Data Innovation Application Laboratory, Suzhou, Jiangsu, China; ^3^ Information Department, Affiliated Kunshan Hospital of Jiangsu University, Suzhou, Jiangsu, China; ^4^ Kunshan Municipal Health and Family Planning Information Center, Suzhou, Jiangsu, China

**Keywords:** osteoporosis, osteoporotic fracture, serum uric acid, triglyceride-glucose index, older people

## Abstract

**Background:**

The triglyceride-glucose index (TyG) has been linked to metabolic disorders, yet its association with serum uric acid (SUA) in elderly osteoporosis patients remains unclear. This study aimed to determine whether TyG independently correlates with SUA levels in osteoporotic fracture (OPF) patients aged >50 years.

**Methods:**

This was a retrospective cross-sectional analysis of data from 2,152 OPF patients from the Affiliated Kunshan Hospital of Jiangsu University database hospitalized between January 2017 and July 2022. Baseline TyG was the exposure variable, whereas SUA levels were the study outcome. When analyzing this relationship, adjustments were made for age, gender, BMI, and various baseline clinical and laboratory parameters, followed by the fitting of separate univariate and multivariate linear regression models. Non-linear association analyses were also conducted with the generalized additive model (GAM). This relationship was further characterized through smooth curve fitting, univariate analysis, and threshold effect analyses.

**Results:**

After adjusting for confounders, an S-shaped relationship between TyG and SUA levels was identified and fitted to a two-piecewise linear regression model with inflection points at 6.34 and 8.09 (*P*-value for LRT < 0.01). Significant positive correlation was observed within the range 6.34–8.09 (β = 27.73, 95% CI: 18.72–36.75, *P* < 0.01), whereas no significant association was found below or above this range.

**Conclusions:**

This study demonstrates a non-linear S-shaped relationship between TyG and SUA levels in OPF patients, with a significant correlation observed only within a specific TyG range. These findings provide novel insights into the metabolic implications of TyG in elderly individuals with osteoporosis.

## 1 Introduction

Osteoporotic fractures (OPFs) are a common skeletal condition affecting middle-aged and older adults (1). They are characterized as low-energy or fragility fractures and represent the severe stage of osteoporosis (OP), associated with high rates of morbidity, disability, mortality, and significant medical costs (2). Globally, one OPF occurs every three seconds, and approximately 50% of women and 20% of men will experience their first OPF after the age of 50, and half of the individuals who sustain one fracture are likely to suffer another (3). In China alone, there were an estimated 2.33 million OPF cases in 2010, a figure projected to increase to 5.99 million by 2050 (4). Therefore, OPFs are not just a threat to public health, but also a pressing social issue, highlighting the urgency required to prevent these fractures and to monitor patients.

Insulin resistance (IR) has been implicated in pathological bone turnover and disruptions in bone homeostasis (5). While the hyperinsulinemic euglycemic glucose clamp (HEGC) approach remains a “gold standard” for IR assessment, it is expensive, complex, and entails multiple rounds of blood collection that limit its clinical feasibility (6). To address these challenges, the triglyceride-glucose (TyG) index, calculated as ln [triglyceride (TG) (mg/dL) × fasting blood glucose (FBG) (mg/dL)/2] (5), has emerged as a promising alternative biomarker for IR (7). Recent research has linked the TyG index to various conditions, including coronary artery disease, hypertension, ischemic stroke, heart failure, liver fibrosis, and kidney stones (6, 8–11). Additionally, mounting evidence highlights a strong association between IR and levels of serum uric acid (SUA) (12–14).

SUA is the final product produced through the metabolism of purines or the catabolic processing of purine nucleotides (15). It is known to exhibit potent extracellular antioxidant properties, scavenging oxygen free radicals generated by oxidative stress and preventing oxidative damage (16). Humans, however, lack the uricase enzyme, which converts uric acid into highly soluble compounds. As a result, urate remains in circulation, leading to elevated basal SUA levels (17). High SUA levels are strongly associated with diabetes, hypertension, obesity, renal function decline, and cardiovascular diseases (18–20). Hyperuricemia contributes to IR by promoting mitochondrial oxidative stress, inducing inflammation, and disrupting insulin signaling pathways (21). An observational study of 5,012 healthy adolescents followed over 15 years supports the use of SUA levels as a simple indicator for predicting the future onset of type 2 diabetes and IR (22). Furthermore, elevated SUA levels can induce inflammation and oxidative stress, increasing the activity of bone-resorbing cells, inhibiting osteoblast function, and ultimately causing bone loss and osteoporosis (23).

Previous research has predominantly focused on the relationship between SUA and the TyG index in younger populations, including children and college students (14, 24). However, there is limited evidence examining this relationship in older adults, particularly those with OPFs. Therefore, this study focused at length on the independent association between TyG and SUA among patients 50+ years of age with OPFs.

## 2 Materials and methods

### 2.1 Study design and population

**Figure 1:**
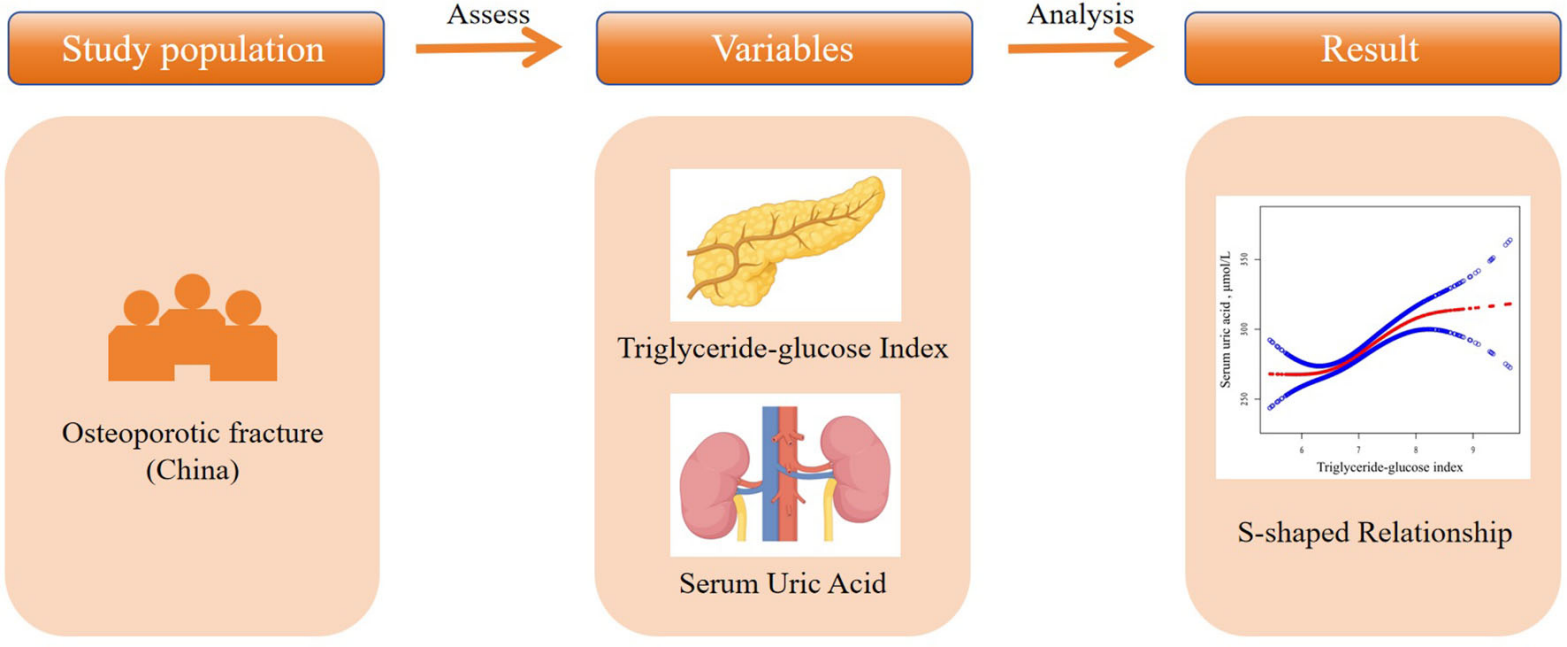
Graphical Abstract.

**Figure 2:**
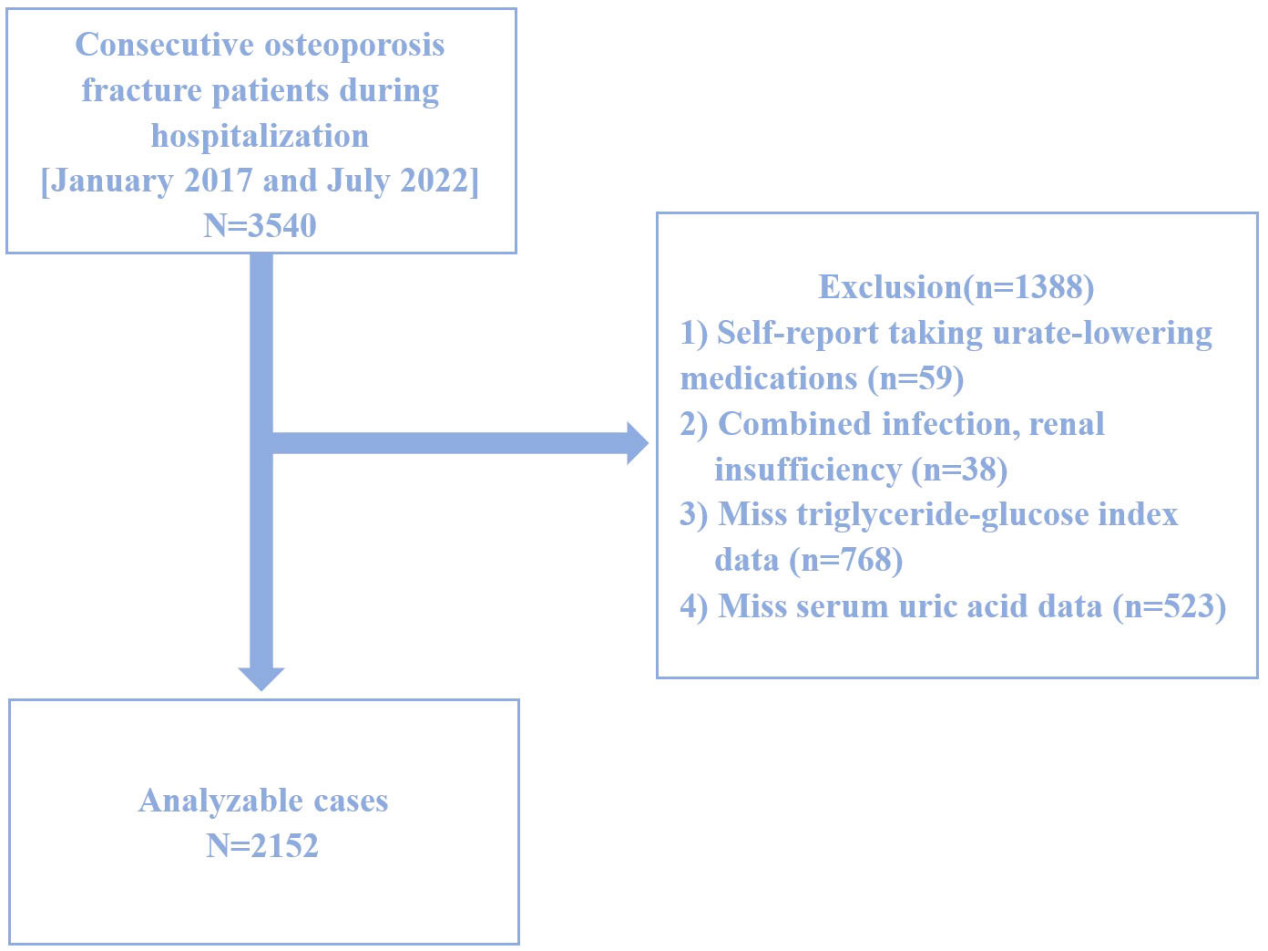
Study flow chart.

This study is a retrospective cross-sectional analysis based on data collected between January 2017 and July 2022 at the Affiliated Kunshan Hospital of Jiangsu University, Suzhou, China. Graphical abstract is shown in **Figure 1**. A total of 2,152 hospitalized patients with newly diagnosed OPF aged over 50 were included. The diagnosis of OPF was based on the presence of fragility fractures without other metabolic bone disorders and, in some cases, a normal bone mineral density (BMD) T-score (25). In 2013, the International Osteoporosis Foundation (IOF) proposed a more concise definition of OPF: a fracture resulting from low-energy trauma during routine activities (for example, a fall from standing height) (26). OPFs are the greatest clinical risk associated with OP, and their diagnosis is based on the presence of this condition (27). OP, in turn, can be diagnosed because patients exhibit fragility fractures without other metabolic bone disorders, or if they exhibit a normal bone mineral density (T-score). OP can also be diagnosed in those patients with a T-score ≤ -2.5 even if fractures are absent (28). Patients were excluded from the present study if (1) they self-reported using urate-lowering medications (n=59), (2) exhibited comorbid infections or renal insufficiency (n=38), (3) were missing TyG index data (n=768), or (4) were missing SUA data (n=523). A schematic diagram of the patient selection process is presented in **Figure 2**. The study adheres to the principles of the Helsinki Declaration and was approved by the Ethics Committee of Kunshan Hospital at Jiangsu University (Approval No. 2021-06-016-K01). All patients provided written informed consent, and their identities were anonymized to ensure objectivity.

### 2.2 Dependent variables

Fasting blood samples were collected within 24 hours of admission by trained personnel using standardized procedures and the same equipment. The TyG index, the dependent variable, was calculated as Ln[fasting triglycerides (mg/dl) × fasting glucose (mg/dl)/2] (29). Fasting blood glucose levels were measured using the hexokinase method, while serum triglycerides were quantified through enzymatic assays.

### 2.3 Exposure variables

SUA levels, the exposure variable, were determined using an enzymatic colorimetric method performed between January 2017 and July 2022. Measurements were obtained using the Beckman AU5800 biochemical analyzer, operated by the same experienced personnel under standardized protocols. Quality control procedures were conducted daily before data collection.

### 2.4 Covariates

Potential covariates included age, gender, BMI (body mass index), Charlson comorbidity index (CCI), creatinine (Cr), hemoglobin, calcium, lymphocyte count, monocyte count, and parathyroid hormone (PTH) levels. All blood samples were collected after fasting.

### 2.5 Statistical analyses

Continuous and categorical variables are presented as means ± standard deviations (SDs), medians (Q1, Q3), or frequencies (%), as appropriate. Fisher’s exact test or Pearson’s chi-squared test was used for categorical variables, while t-tests and Mann–Whitney U tests were employed for continuous variables. Linear regression models were used to evaluate the association between TyG and SUA in hospitalized OPF patients.

Generalized estimating equations (GEE) were applied to examine the independent relationship between TyG and SUA, with adjustments for covariates. Three models were developed, including minimally and fully adjusted models. Initially, variance inflation factor (VIF) analyses were employed to detect covariance collinearity, and whether or not covariates were adjusted for was determined based on: (1) changes in matched odds ratios (ORs) ≥ 10% when adding the covariates to the unadjusted model or removing it from the fully adjusted model, and (2) covariates that met criterion 1 or exhibited a *P* < 0.1 in univariate analyses (30). The three established models using this approach included Model 1 (unadjusted), Model 2 (adjusted for age, gender, BMI, CCI, Cr), and Model 3 (further adjusted for hemoglobin, calcium, lymphocytes, monocytes, PTH).

Generalized additive models (GAMs) were employed to detect non-linear relationships, and two-piecewise linear regression models identified threshold effects. The inflection point was automatically calculated using a recursive maximum likelihood method (31). Subgroup analyses were performed to evaluate variations across patient subgroups, with the likelihood ratio test (LRT) assessing interactions and modifications.

All statistical analyses were conducted using EmpowerStats (www.empowerstats.com, X&Y Solutions, Inc., MA, USA) and R 3.6.3 (www.r-project.org). *P* < 0.05 was considered significant.

## 3 Results

### 3.1 Patients characteristics

The baseline data for 2,152 patients with OPFs hospitalized between January 2017 and July 2022 stratified into SUA quartiles are compiled in **Table 1**. This overall patient population (31.89% male, 68.11% female) exhibited a mean age of 69.20 ± 11.24 years, a mean SUA level of 284.84 ± 92.24 μmol/L, and a mean TyG of 6.92 ± 0.60. When patients were assessed following classification into TyG tertiles (< 6.62, 6.62–7.12, and > 7.12), their SUA, hemoglobin, calcium, lymphocyte, UN, HDL, LDL, and apolipoprotein B levels differed significantly. Notably, patients in higher TyG tertiles were more likely to present with elevated SUA levels (Low: 263.65 ± 80.35 μmol/L; Middle: 277.39 ± 86.51 μmol/L; High: 303.73 ± 100.59 μmol/L, *P* < 0.01).

**Table 1:** Patient characteristics based on TyG quartiles.

Characteristics	Total	Mean + SD / N(%)			*P*-value	*P*-value*
		Q1 (<6.62)	Q2 (6.62-7.12)	Q3 (>7.12)		
N	2152	717	717	718		
Gender, N (%)					0.90	-
Female	2411 (68.11%)	485 (67.64%)	478 (66.67%)	486 (67.69%)		
Male	1129 (31.89%)	232 (32.36%)	239 (33.33%)	232 (32.31%)		
Age 4 quantiles, y					0.96	-
Q1(50-60y)	807 (22.80%)	167 (23.29%)	155 (21.62%)	171 (23.82%)		
Q2(60-68y)	852 (24.07%)	175 (24.41%)	175 (24.41%)	167 (23.26%)		
Q3(68-78y)	961 (27.15%)	191 (26.64%)	203 (28.31%)	195 (27.16%)		
Q4(78-87y)	920 (25.99%)	184 (25.66%)	184 (25.66%)	185 (25.77%)		
BMI categorical, N(%)					0.32	-
≤24 kg/m2	2190 (61.86%)	443 (61.79%)	452 (63.04%)	437 (60.86%)		
24-28 kg/m^2^	1105 (31.22%)	234 (32.64%)	218 (30.41%)	222 (30.92%)		
>28 kg/m^2^	245 (6.92%)	40 (5.58%)	47 (6.56%)	59 (8.22%)		
SUA, μmol/L	284.84 ± 92.24	263.65 ± 80.35	277.39 ± 86.51	303.73 ± 100.59	<0.01	<0.01
Cr, μmol/L	66.64 ± 40.32	63.11 ± 19.47	64.99 ± 21.67	68.32 ± 46.69	<0.01	0.35
Hemoglobin, g/L	125.73 ± 18.37	124.09 ± 17.90	125.51 ± 18.34	128.72 ± 17.02	<0.01	<0.01
Calcium, mmol/L	2.21 ± 0.13	2.18 ± 0.13	2.20 ± 0.12	2.24 ± 0.13	<0.01	<0.01
Lymphocyte count, × 109/L	1.24 ± 0.53	1.16 ± 0.53	1.25 ± 0.53	1.36 ± 0.56	<0.01	<0.01
Monocyte count, × 109/L	0.51 ± 0.30	0.51 ± 0.25	0.48 ± 0.23	0.49 ± 0.45	0.46	0.07
PTH, pmol/L	14.62 ± 10.34	14.10 ± 11.97	15.29 ± 10.35	14.30 ± 8.41	0.07	<0.01
UN, μmol/L	6.07 ± 3.35	5.81 ± 1.96	6.06 ± 2.22	6.30 ± 3.00	<0.01	<0.01
BTX, ug/L	0.52 ± 0.26	0.53 ± 0.27	0.55 ± 0.26	0.52 ± 0.25	0.57	0.71
P1NP, ug/L	56.04 ± 24.88	54.51 ± 25.53	57.25± 25.69	56.83 ± 25.65	0.55	0.33
HDL, mmol/L	1.34 ± 0.31	1.44 ± 0.30	1.36 ± 0.30	1.24 ± 0.30	<0.01	<0.01
LDL, mmol/L	2.55 ± 0.76	2.21 ± 0.64	2.56 ± 0.69	2.88 ± 0.79	<0.01	<0.01
AST, U/L	26.40 ± 21.77	25.82 ± 19.13	24.04 ± 12.99	24.57 ± 12.15	0.07	0.20
Apolipoprotein A, g/L	1.22 ± 0.24	1.22 ± 0.24	1.22 ± 0.25	1.21 ± 0.24	0.80	0.93
Apolipoprotein B, g/L	0.81 ± 0.22	0.71 ± 0.19	0.81 ± 0.20	0.91 ± 0.22	<0.01	<0.01
CCI score categorical, N (%)					0.53	-
0	3163 (89.35%)	650 (90.66%)	642 (89.54%)	644 (89.69%)		
1	296 (8.36%)	57 (7.95%)	58 (8.09%)	59 (8.22%)		
2	58 (1.64%)	7 (0.98%)	13 (1.81%)	7 (0.98%)		
3	15 (0.42%)	2 (0.28%)	2 (0.28%)	6 (0.84%)		
4	5 (0.14%)	0 (0.00%)	1 (0.14%)	1 (0.14%)		
5	1 (0.03%)	0 (0.00%)	1 (0.14%)	0 (0.00%)		
7	1 (0.03%)	1 (0.14%)	0 (0.00%)	0 (0.00%)		
8	1 (0.03%)	0 (0.00%)	0 (0.00%)	1 (0.14%)		
ASA					0.54	-
1	317 (8.96%)	68 (9.48%)	56 (7.81%)	60 (8.36%)		
2	2382 (67.29%)	478 (66.67%)	492 (68.62%)	486 (67.69%)		
3	829 (23.42%)	171 (23.85%)	166 (23.15%)	171 (23.82%)		
4	12 (0.34%)	0 (0.00%)	3 (0.42%)	1 (0.14%)		

TyG, triglyceride-glucose; SD, standard deviation; Q1, first quartile; Q2, second quartile; Q3, third quartile; SUA, serum uric acid; Cr, creatinine; PTH, parathyroid hormone; UN, urea nitrogen; BTX, botulinum toxin; P1NP, N-terminal propeptide of type I procollagen; HDL, high-density lipoproteins; LDL, low-density lipoproteins; AST, aspartate aminotransferase; BMI, body mass index; CCI, Charlson comorbidity index; ASA, American Society of Anesthesiologists.

P-value*: Kruskal Wallis Rank Test for continuous variables, Fisher Exact for categorical variables with Expects < 10.

### 3.2 Univariate analyses

Univariate analytical efforts revealed that SUA levels were associated with Cr, hemoglobin, calcium, monocyte, PTH, UN, BTX, P1NP, HDL, AST, apolipoprotein A, and apolipoprotein B levels (**Table 2**), but not with any other analyzed variables.

**Table 2:** Univariate analyses of factors associated with SUA levels.

Characteristics	Statistics	β^a^ (95% CI)	*P*-value
Age 4 quantiles, y			
Q1(50-60y)	493 (22.91%)	Reference	Reference
Q2(60-68y)	517 (24.02%)	4.497 (-6.71, 15.70)	0.43
Q3(68-78y)	589 (27.37%)	2.75 (-8.12, 13.62)	0.62
Q4(78-87y)	553 (25.70%)	19.03 (8.00, 30.05)	<0.01
Gender, N(%)			
Female	1449 (67.33%)	Reference	Reference
Male	703 (32.67%)	6.48 (-1.72, 14.68)	0.12
BMI categorical, N(%)			
≤24 kg/m2	1332 (61.90%)	Reference	Reference
24-28 kg/m^2^	674 (31.32%)	-11.01 (-19.43, -2.59)	0.01
>28 kg/m^2^	146 (6.78%)	9.35 (-6.184, 24.88)	0.24
CCI score categorical, N (%)			
0	1936 (89.96%)	Reference	Reference
1	174 (8.09%)	-9.29(-23.40, 4.82)	0.20
2	27 (1.26%)	-14.07 (-48.62, 20.48)	0.43
3	10 (0.47%)	-1.29 (-57.82, 55.23)	0.96
4	2 (0.09%)	167.71 (41.57, 293.84)	<0.01
5	1 (0.05%)	26.71 (-151.63, 205.04)	0.77
7	1 (0.05%)	113.71 (-64.63, 292.04)	0.21
8	1 (0.05%)	35.71 (-142.63, 214.04)	0.70
Cr, μmol/L	65.47 ± 31.84	1.05 (0.94, 1.17)	<0.01
Hemoglobin, g/L	126.11 ± 17.86	0.63 (0.42, 0.85)	<0.01
Calcium, mmol/L	2.21 ± 0.13	99.24 (69.94, 128.53)	<0.01
Lymphocyte count, × 109/L	1.26 ± 0.55	6.70 (-0.38, 13.78)	0.06
Monocyte count, × 109/L	0.49 ± 0.33	31.43 (19.65, 43.21)	<0.01
PTH, pmol/L	14.56 ± 10.36	1.90 (1.54, 2.27)	<0.01
UN, μmol/L	6.06 ± 2.44	12.43 (10.94, 13.92)	<0.01
BTX, ug/L	0.53 ± 0.26	-49.94 (-77.80, -22.07)	<0.01
P1NP, ug/L	56.26 ± 25.61	-0.33 (-0.61, -0.05)	0.02
HDL, mmol/L	1.35 ± 0.31	-54.64 (-66.83, -42.45)	<0.01
LDL, mmol/L	2.55 ± 0.76	4.04 (-1.01, 9.10)	0.12
AST, U/L	24.81 ± 15.09	0.50 (0.25, 0.75)	<0.01
Apolipoprotein A, g/L	1.22 ± 0.24	-32.79 (-48.71, -16.87)	<0.01
Apolipoprotein B, g/L	0.81 ± 0.22	20.42 (2.98, 37.85)	0.02
ASA			
1	184 (8.55%)	Reference	Reference
2	1456 (67.66%)	5.15 (-8.80, 19.11)	0.47
3	508 (23.61%)	13.73 (-1.61, 29.08)	0.08
4	4 (0.19%)	-39.67 (-129.84, 50.44)	0.39
TYG	6.92 ± 0.60	29.70 (23.39, 36.00)	<0.01

a Dependent variable SUA, as a result of univariate analyses for SUA.

SUA, serum uric acid; TyG, triglyceride-glucose; SD, standard deviation; Q1, first quartile; Q2, second quartile; Q3, third quartile; Q4, fourth quartile; BMI, body mass index; CCI, Charlson comorbidity index; Cr, creatinine; PTH, parathyroid hormone; UN, urea nitrogen; BTX, botulinum toxin; P1NP, N-terminal propeptide of type I procollagen; HDL, high-density lipoproteins; LDL, low-density lipoproteins; AST, aspartate aminotransferase; ASA, American Society of Anesthesiologists.

### 3.3 Examining the relationship between TyG and SUA levels

The interplay between TyG and SUA levels among OPF patients was next explored through the development of three models (**Table 3**). In the unadjusted Model 1, a strong association was observed (β = 29.70, 95% CI: 23.39 to 36.00, *P* < 0.01). This association remained significant in Model 2, which was adjusted for age, gender, BMI, CCI, and Cr (β = 25.64, 95% CI: 19.75 to 31.53, *P* < 0.01). Similarly, in the fully adjusted Model 3, which included additional covariates such as hemoglobin, calcium, lymphocytes, monocytes, and PTH, a positive relationship persisted (β = 20.68, 95% CI: 14.75 to 26.60, *P* < 0.01).

**Table 3:** Association between TyG and SUA levels in different models.

	Model 1^a^ N=2142		Model 2^b^ N=2142		Model 3^c^ N=2142	
	β(95%CI)	*P*-value	β(95%CI)	*P*-value	β(95%CI)	*P*-value
TyG per 1 increase	29.70 (23.39, 36.00)	<0.01	25.64 (19.75, 31.53)	<0.01	20.68 (14.75, 26.60)	<0.01
TYG quartile						
Q1(<6.620)	Reference	Reference	Reference	Reference	Reference	Reference
Q2(6.620-7.119)	13.73 (4.46, 23.00)	<0.01	11.71 (3.09, 20.33)	<0.01	7.90 (-0.52, 16.33)	0.07
Q3(>7.119)	40.07 (30.81, 49.34)	<0.01	35.01 (26.38, 43.65)	<0.01	28.45 (19.80, 37.10)	<0.01

a No adjustment.

b Adjusted for age, gender, BMI, CCI, Cr.

c Adjusted for age, gender, BMI, CCI, Cr, hemoglobin, calcium, lymphocyte, monocyte, PTH.

TyG, triglyceride-glucose; SUA, serum uric acid; Q1, first quartile; Q2, second quartile; Q3, third quartile.

When patients were grouped into tertiles based on TyG levels, SUA levels were found to be significantly higher in the second and third tertiles compared to the first. Specifically, the average SUA levels in tertiles Q2 and Q3 were 7.90 and 28.45 units higher, respectively, in Model 3. This upward trend in SUA levels across TyG tertiles was consistent across all three models.

To ensure the robustness of Model 3, subgroup analyses were conducted by stratifying patients based on factors such as age, gender, BMI, CCI, Cr, hemoglobin, calcium, lymphocyte count, monocyte count, and PTH. The analyses, adjusted for the remaining covariates not used for stratification, revealed a consistent relationship between TyG and SUA levels across all subgroups without any significant interaction effects (all *P* > 0.05, **Supplementary Table S1**). Notably, the positive association between TyG and SUA was consistent across both male and female subgroups.

### 3.4 Spline smoothing and threshold analyses

**Figure 3:**
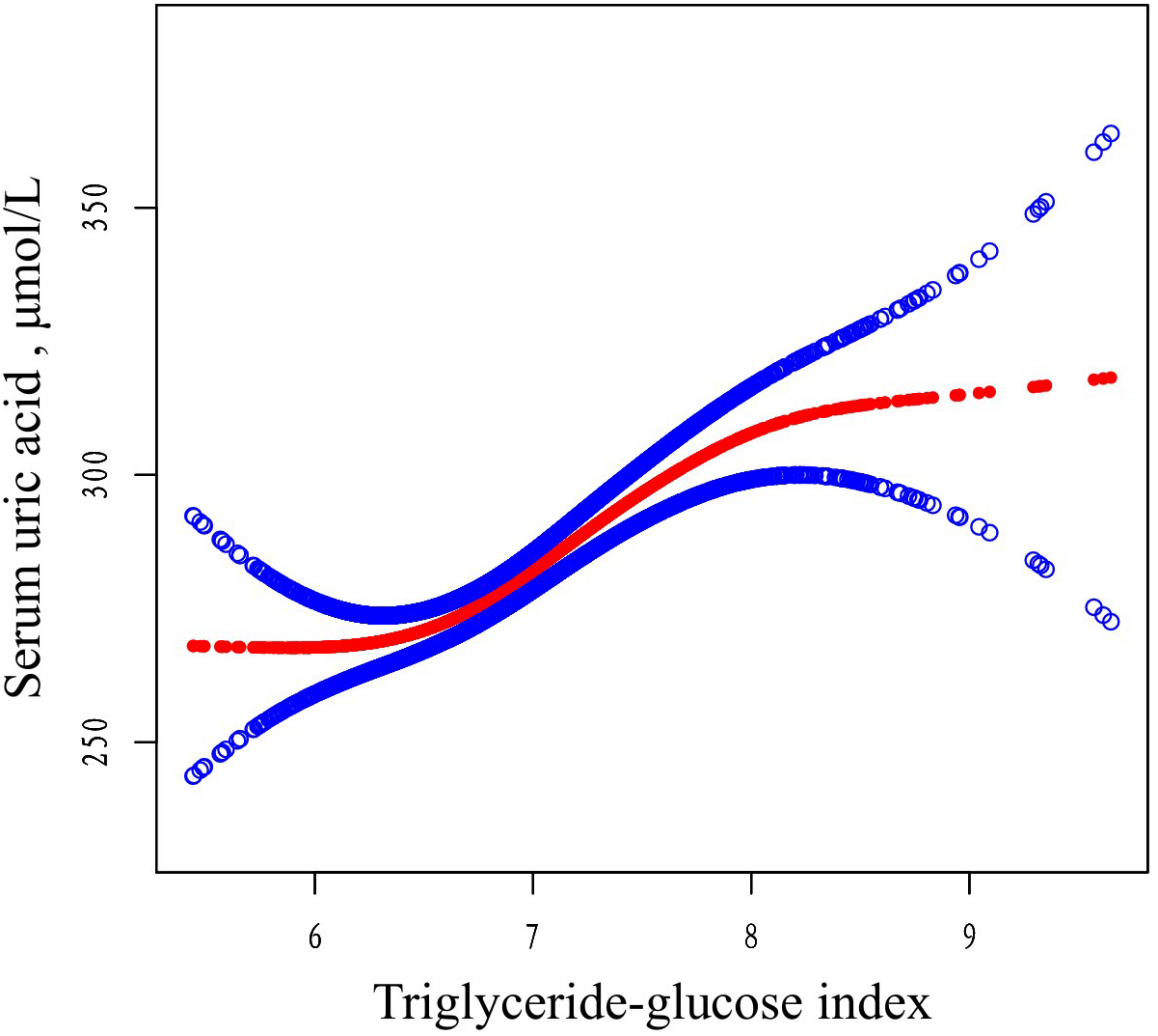
The relationship between TyG and SUA. Adjusted smoothed curves corresponding to the relationship between TyG and SUA. A generalized additive model revealed a thresholded non-linear relationship between TyG and SUA in OP patients. The upper and lower curves represent the range of the 95% confidence interval, and the middle curve represents the correlation between FAR and SIRI. Models were adjusted for age, gender, BMI, CCI, Cr, hemoglobin, calcium, lymphocyte, monocyte, PTH. The red curve in Model 3 exhibited two inflection points (K) at 6.34 and 8.09. TyG, triglyceride-glucose; SUA, serum uric acid; BMI, body mass index; CCI, Charlson comorbidity index; Cr, uric acid; PTH, parathyroid hormone.

A generalized additive model (GAM) was used to explore the potential nonlinear relationship between TyG and SUA levels. As shown in **Figure 3**, a clear nonlinear relationship was observed after adjusting for covariates (age, gender, BMI, CCI, Cr, hemoglobin, calcium, lymphocyte count, monocyte count, and PTH). To identify possible inflection points, a two-piecewise linear regression model was applied. This analysis revealed two inflection points for TyG at 6.34 and 8.09, respectively (*P* < 0.01 for the log-likelihood ratio). A significant positive association between TyG and SUA levels was observed within the range of 6.34 to 8.09 (β = 27.73, 95% CI: 18.72 to 36.75, *P* < 0.01). However, outside this range, the association was not significant, with β values of -11.30 (95% CI: -39.30 to 16.69, *P* = 0.43) for TyG < 6.34 and -34.10 (95% CI: -78.95 to 10.75, *P* = 0.14) for TyG > 8.09 (**Table 4**).

**Table 4:** Threshold effect analysis of the relationship between TyG and SUA levels using piece-wiselinear regression.

Outcome	Model 3^a^	
	SUA β(95%CI)	*P*-value
Model A^b^		
One line effect	20.68 (14.75, 26.60)	<0.01
Model B^c^		
TyG turning point (K)	6.34, 8.09	
< 6.34	-11.30 (-39.30, 16.69)	0.43
6.34-8.09	27.73 (18.72, 36.75)	<0.01
> 8.09	-34.10 (-78.95, 10.75)	0.14
LRT test^d^		<0.01

a Adjusted for age, gender, BMI, CCI, Cr, hemoglobin, calcium, lymphocyte, monocyte, PTH.

b Linear analysis, *P*-value < 0.05 indicates a linear relationship.

c Three-piecewise linear analysis.

d *P*-value < 0.05 means Model B is significantly different from Model A, which indicates a non-linear relationship.

TyG, triglyceride-glucose; SUA, serum uric acid; LRT, logarithmic likelihood ratio test.

## 4 Discussion

This study identified an S-shaped association between TyG and SUA levels in a cross-sectional analysis of 2,152 hospitalized patients with OPF. Using a two-piecewise linear regression model, two inflection points were determined at 6.34 and 8.09. Within this range, a significant positive correlation was observed between TyG and SUA levels, with SUA levels remaining within normal physiological limits. These findings suggest that the TyG index may serve as a novel biomarker for evaluating the metabolic equilibrium of SUA levels.

Previous epidemiological studies have also examined the relationship between TyG and SUA levels, and the present results are consistent with these findings. For example, a study involving 1,700 children and adolescents with short stature in China recruited from the Affiliated Hospital of Jining Medical University in China between March 2013 and April 2021 reported a nonlinear association between TyG and SUA levels. A positive correlation was observed when SUA levels exceeded 6.55 mg/dL, while no significant association was found below this threshold (24). Similarly, another study involving 23,411 young adults (17–20 years old) in China recruited from Qingdao University from September 2017 to October 2019 found that lipid accumulation product (LAP), TyG, and their derivatives were strongly associated with SUA levels, suggesting their utility as sensitive indicators for predicting hyperuricemia (HUA) (14). While these past studies focused on younger populations, the present research extends these findings to an older population, particularly those over 50 years old with OPF.

Zhang et al. reported a positive association between TyG and hyperuricemia in a general health checkup/community population, focusing on linear association and predictive performance, but did not examine osteoporotic patients or test for nonlinear inflection points (32). Song et al. investigated the relationships between TyG and multiple metabolic indicators and conducted subgroup analyses; however, their analysis relied primarily on conventional multivariable linear/Logistic regression and did not systematically apply spline or piecewise regression to locate nonlinear breakpoints (33). Wang et al. emphasized the utility of TyG in metabolic syndrome and cardiovascular risk assessment but did not evaluate whether bone metabolic status modifies the TyG–SUA relationship (34). Hu et al. analyzed large cohorts and reported a robust positive association between TyG and metabolic abnormalities, discussing mechanisms such as insulin resistance, yet they did not provide empirical analyses of inflection points or bidirectional effects within specific subgroups (35).In contrast, the present study makes the following distinct contributions: it focuses on a clinical cohort of patients with osteoporosis; it employs smoothing splines and breakpoint detection to precisely characterize and quantify an S-shaped (nonlinear) TyG–SUA relationship; and it adjusts models for osteoporosis-related covariates while performing subgroup and sensitivity analyses. These methodological and population-specific differences support the interpretation that, within an osteoporotic population, bone metabolism, and interactions with uric acid handling may underlie the observed nonlinear association.Zhang et al.

Our analysis provides the first evidence of an S-shaped correlation between TyG and SUA in older adults with OPF. Although the association between the TyG index and SUA is well-supported by previous research, the underlying mechanisms remain unclear. The TyG index, calculated based upon fasting plasma glucose and triglyceride levels, is a reliable marker for metabolic syndrome and insulin resistance (IR) (36, 37). The link between TyG and SUA may be mediated by IR, as studies have shown that IR promotes the production of SUA through increased glycolysis intermediates which are transferred to 5-phosphoribose and phosphoric acid ribose pyrophosphate under IR conditions, triggering SUA production (38). High IR-related insulin levels also promote Na^+^-H^+^ exchange in the renal tubules, enhance the excretion of H^+^, and favor the reabsorption of UA (39), while renin-angiotensin system activity in response to hyperinsulinemia leads to a drop in blood flow in the kidneys, greater urate reabsorption, and xanthine oxidase production that contribute to higher levels of SUA output (40), McCormick et al. also demonstrated that it positively and causally affects SUA concentrations (41). Epidemiological efforts have also offered support for the link between IR and SUA, with the compensatory hyperinsulinemia following IR reducing the excretion of uric acid via renal tubular sodium reabsorption and thereby elevating levels of SUA (42–44). Conversely, higher levels of UA can promote IR through reduced nitric oxide bioavailability and greater oxidative stress in the mitochondria (45). These findings underscore the bidirectional relationship between SUA and IR and highlight the importance of monitoring IR status in elderly patients to manage SUA levels and prevent metabolic disorders, including osteoporosis.

The clinical implications of this study are significant, offering evidence-based recommendations for managing bone metabolic disorders in elderly patients. The TyG index provides a simple and cost-effective method for assessing SUA levels and identifying individuals at risk, particularly among populations with OPFs. The TyG, as a simple, cost−effective, and readily obtainable measure, can be used to assess SUA levels and to identify high−risk individuals—an application that is particularly relevant in populations at elevated risk of fracture. Kahaer et al. demonstrated a significant link between the TyG and HUA such that it could be leveraged to screen for HUA risk among the Chinese Xinjiang population (46). Moreover, da Silva et al. corroborated the linear association between the TyG and HUA among the general Chinese populace, underscoring the predictive value of this index (47). These past reports emphasize the value of analyzing the TyG when seeking to gauge HUA risk and prevent this condition. On this basis, the present study expands the evidence base and offers clinically meaningful insights in several respects. First, we focus on elderly patients with OP who are at elevated fracture risk—a vulnerable subgroup in whom metabolic dysregulation may exert disproportionately adverse effects on bone health, functional recovery, and survival outcomes. Second, by identifying an S−shaped association and quantifying threshold intervals, we propose that TyG values between 6.34 and 8.09 are associated with relatively normal SUA levels, whereas TyG values below or above this interval may indicate an increased risk of metabolic imbalance. These empirically derived cutoffs may assist clinicians in identifying patients who warrant closer biochemical surveillance or earlier metabolic intervention.For elderly patients already burdened by OP and fracture risk, maintaining SUA within the range identified in this study may contribute to improved overall health outcomes and could potentially reduce mortality risk associated with the concurrence of metabolic and skeletal disorders. From a clinical practice perspective, this implies intensified monitoring of TyG and SUA, optimization of glucose and lipid metabolic control, careful evaluation and adjustment of medications that affect SUA homeostasis, and targeted lifestyle or pharmacologic interventions tailored to this vulnerable population. Finally, these proposed thresholds and the elucidation of a nonlinear TyG–SUA relationship require prospective validation and may ultimately be incorporated into clinical risk−stratification tools to determine whether TyG−based interventions can improve bone−related outcomes and survival in older adults.

There are several key strengths to this study. For one, rigorous screening of the study population was performed, and three models adjusted for many covariates (age, gender, BMI, CCI, Cr, hemoglobin, calcium, lymphocytes, monocytes, and PTH) were used to probe the association between TyG and SUA levels. Generalized linear model and GAM approaches were also employed to respectively assess the linearity and non-linearity of this relationship. GAM approaches are well-suited to non-parametric smoothing to help fit regression splines to datasets, providing a more effective means of examining the interplay between average TyG and SUA levels.

There are also some limitations to the study. For one, although we observed an association between TyG and SUA, the retrospective cross−sectional design of the study precludes any inference of causality. In addition, certain covariates that were not measured or were insufficiently controlled in this study — for example, detailed dietary intake, alcohol consumption, smoking status, physical activity levels, inflammatory markers, and specific medications such as diuretics, statins, and bisphosphonates — may have influenced the analytical results, and residual confounding cannot be excluded. Future studies should collect more comprehensive information on lifestyle factors and medication use, and incorporate additional biochemical markers and repeated measurements to improve confounder control and to ensure the robustness and generalizability of the findings. Third, this study was conducted at a single center in China with a relatively small sample size; therefore, the extent to which our findings can be generalized to other ethnic groups and geographic populations remains uncertain. In addition, this study did not use an independent external validation cohort, so the observed associations require verification in other populations or regions to confirm their robustness and generalizability. Finally, these limitations together emphasize the importance of conducting additional multi−center, large−scale cohort studies and randomized controlled trials that recruit more diverse patient populations and examine additional biochemical indicators to ensure the reliability and external validity of the results.

## 5 Conclusions

In summary, this analysis of 2,152 hospitalized OPF patients revealed a marked S-shaped association between TyG and SUA levels, with this relationship being particularly significant in the TyG values ranging from 6.34 to 8.09. The TyG may thus be a valuable index for the assessment of SUA-related metabolic disturbances among elderly individuals.

## Data Availability

The original contributions presented in the study are included in the article/**Supplementary Material**. Further inquiries can be directed to the corresponding authors.
