# Supplementary material for: Exploring the S-shaped relationship between triglyceride-glucose index and serum uric acid levels in individuals with osteoporotic fracture
Source: Front Endocrinol (Lausanne). 2025 Oct 24;16:1639818. doi: 10.3389/fendo.2025.1639818 (PMC12591983; doi:10.3389/fendo.2025.1639818)
Supplement: Supplementary file 1 [file Table1.docx]

Table S1. Subgroup Analyses Exploring the Association Between TyG and SUA levels

|  | N | β (95% CI) | *P-*value for interaction |
| --- | --- | --- | --- |
| Gender |  |  | 0.40 |
| Female | 1449 | 27.84 (20.20, 35.48) |  |
| Male | 703 | 33.68 (22.52, 44.84) |  |
| Age tertile |  |  | 0.22 |
| Low | 714 | 23.74 (12.92, 34.57) |  |
| Middle | 682 | 27.18 (15.90, 38.46) |  |
| High | 756 | 36.73 (26.11, 47.34) |  |
| BMI categorical |  |  | 0.13 |
| ≤24 kg/m^2^ | 1332 | 31.05 (23.04, 39.05) |  |
| 24-28 kg/m^2^ | 674 | 22.60 (11.57, 33.62) |  |
| >24 kg/m^2^ | 146 | 51.24 (24.07, 78.40) |  |
| CCI |  |  | 0.43 |
| 0 | 1936 | 29.30 (29.67, 35.93) |  |
| 1 | 174 | 38.16 (15.10, 61.22) |  |
| 2 | 27 | 22.43 (-37.92,82.77) |  |
| 3 | 10 | 40.80 (-37.74,119.35) |  |
| 4 | 2 | -186.87 (-425.09,51.35) |  |
| 5 | 1 | 32.44 (6.89,57.99) |  |
| 7 | 1 | 58.18 (24.95,91.41) |  |
| 8 | 1 | 32.92 (7.97,57.87) |  |
| Cr |  |  | 0.74 |
| Low | 710 | 25.25 (15.21, 35.29) |  |
| Middle | 740 | 29.97 (20.08, 39.86) |  |
| High | 702 | 30.29 (20.27, 40.30) |  |
| Hemoglobin |  |  | 0.14 |
| Low | 686 | 37.46 (25.69, 49.23) |  |
| Middle | 704 | 27.53 (15.90, 39.15) |  |
| High | 752 | 21.98 (12.27, 31.69) |  |
| Calcium |  |  | 0.66 |
| Low | 711 | 29.42 (17.16, 41.68) |  |
| Middle | 680 | 22.07 (10.58, 33.55) |  |
| High | 761 | 27.60 (17.70, 37.50) |  |
| Lymphocyte |  |  | 0.16 |
| Low | 684 | 21.50 (9.31, 33.70) |  |
| Middle | 665 | 37.39 (26.58, 48.19) |  |
| High | 793 | 29.90 (19.31, 40.50) |  |
| Monocyte |  |  | 0.30 |
| Low | 638 | 23.78 (12.15, 35.41) |  |
| Middle | 829 | 30.43 (20.50, 40.35) |  |
| High | 675 | 36.75 (25.31, 48.19) |  |
| PTH |  |  | 0.49 |
| Low | 723 | 23.59 (13.42, 33.76) |  |
| Middle | 720 | 30.09 (19.49, 40.68) |  |
| High | 709 | 32.27 (21.43, 43.10) |  |

Adjusted for age, gender, BMI, CCI, Cr, hemoglobin, calcium, lymphocyte, monocyte, PTH.

Abbreviations: TyG, triglyceride-glucose index; SUA, serum uric acid; BMI, body mass index; CCI, Charlson comorbidity index; Cr, creatinine; PTH, parathyroid hormone.
